# Cellular Mechanisms Mediating Exercise-Induced Protection against Cardiotoxic Anthracycline Cancer Therapy

**DOI:** 10.3390/cells12091312

**Published:** 2023-05-04

**Authors:** Sanela Dozic, Erin J. Howden, James R. Bell, Kimberley M. Mellor, Lea M. D. Delbridge, Kate L. Weeks

**Affiliations:** 1 Central Clinical School, Monash University, Melbourne, VIC 3004, Australia; 2 Baker Heart and Diabetes Institute, Melbourne, VIC 3004, Australia; 3 Department of Cardiometabolic Health, The University of Melbourne, Parkville, VIC 3010, Australia; 4 Department of Anatomy & Physiology, The University of Melbourne, Parkville, VIC 3010, Australia; 5 Department of Microbiology, Anatomy, Physiology & Pharmacology, La Trobe University, Bundoora, VIC 3086, Australia; 6 Department of Physiology, University of Auckland, Auckland 1023, New Zealand

**Keywords:** cardiotoxicity, exercise, cardioprotection, sex differences, exerkines

## Abstract

Anthracyclines such as doxorubicin are widely used chemotherapy drugs. A common side effect of anthracycline therapy is cardiotoxicity, which can compromise heart function and lead to dilated cardiomyopathy and heart failure. Dexrazoxane and heart failure medications (i.e., beta blockers and drugs targeting the renin–angiotensin system) are prescribed for the primary prevention of cancer therapy-related cardiotoxicity and for the management of cardiac dysfunction and symptoms if they arise during chemotherapy. However, there is a clear need for new therapies to combat the cardiotoxic effects of cancer drugs. Exercise is a cardioprotective stimulus that has recently been shown to improve heart function and prevent functional disability in breast cancer patients undergoing anthracycline chemotherapy. Evidence from preclinical studies supports the use of exercise training to prevent or attenuate the damaging effects of anthracyclines on the cardiovascular system. In this review, we summarise findings from experimental models which provide insight into cellular mechanisms by which exercise may protect the heart from anthracycline-mediated damage, and identify knowledge gaps that require further investigation. Improved understanding of the mechanisms by which exercise protects the heart from anthracyclines may lead to the development of novel therapies to treat cancer therapy-related cardiotoxicity.

## 1. Introduction

**Figure 1:**
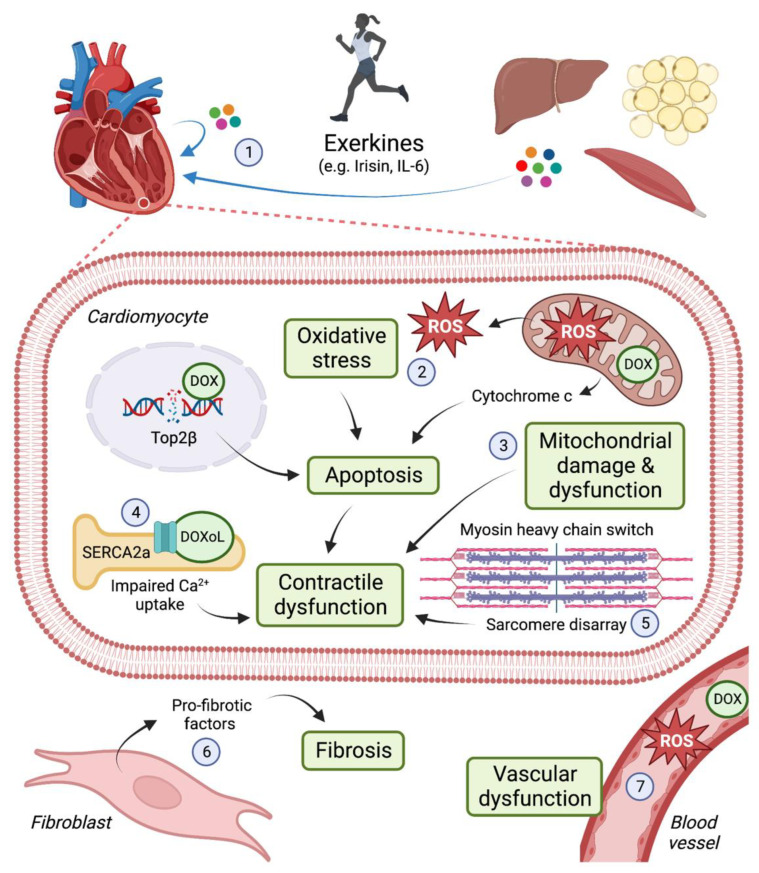
Cellular mechanisms by which exercise protects the heart from the cardiotoxic effects of doxorubicin (DOX) and its metabolite, doxorubicinol (DOXoL). DOX induces cardiotoxicity via multiple mechanisms, including inhibition of topoisomerase 2β (Top2β) which leads to DNA damage and apoptosis; generation of reactive oxygen species (ROS), which causes oxidative stress and mitochondrial damage and dysfunction; contractile impairment arising from inhibition of the sarcomeric reticulum calcium pump (SERCA2a) and changes in myofilament composition and organisation; cardiac fibrosis; and vascular dysfunction. Some of the mechanisms by which exercise may exert cardioprotective effects in settings of DOX therapy include: (1) exerkine release from the heart and other tissues (e.g., skeletal muscle, adipose, liver); (2) upregulation of antioxidants; (3) preservation of mitochondrial structure and function; (4) increased SERCA2a expression; (5) maintenance of myosin heavy chain isoform expression and sarcomere integrity; (6) attenuation of fibrosis; and (7) maintenance of endothelial and smooth muscle cell function. IL-6: interleukin-6. Image created with Biorender.com.

Doxorubicin (DOX) is an anthracycline used to treat various solid tumours and blood cancers, including breast cancer, leukaemia, lymphoma and sarcoma. DOX induces apoptosis of proliferating cells (i.e., cancer cells) by intercalating with DNA and inhibiting the activity of topoisomerase IIα (Top2α) [1,2]. Top2 enzymes induce transient double-stranded breaks in DNA to facilitate unwinding of DNA during transcription and replication. DOX interferes with Top2α-mediated DNA re-ligation, leading to DNA damage and the induction of cell death. Despite the efficacy of anthracyclines as anti-cancer agents, the use of anthracyclines is limited as they can cause irreversible damage to the heart and increase the risk for congestive heart failure (HF) [3]. Anthracycline-associated cardiotoxicity is due in part to inhibition of Top2β, the predominant Top2 isoform expressed in non-proliferative cells, including cardiomyocytes [4,5]. Oxidative stress is another key mechanism by which DOX damages the heart. DOX accumulates in mitochondria leading to the production of reactive oxygen species (ROS), mitochondrial damage and dysfunction [6,7]. As cardiomyocytes are enriched in mitochondria to meet the heart’s constant demand for ATP, this cell type is particularly vulnerable to DOX-induced damage. (See Figure 1).

Anthracycline-associated cardiotoxicity is dose-dependent and can occur acutely (i.e., during treatment) or in the months or years following treatment. Paediatric cancer survivors who received anthracycline chemotherapy are at particularly high risk of cardiovascular complications later in life [8] and cardiovascular disease is the leading non-cancer cause of death amongst survivors of both breast cancer and childhood cancer [9,10]. The first clinical guidelines for identifying and managing cardiovascular risk in cancer patients were published in 2022 [11]. Routine HF medications, including beta blockers and angiotensin-converting enzyme inhibitors (ACEi) or angiotensin receptor blockers (ARBs), have been shown to attenuate anthracycline-associated declines in left ventricular ejection fraction (LVEF, a measure of systolic function), [12] and are therefore recommended for patients at high or very high risk of anthracycline-associated cardiotoxicity [11]. Due to a lack of large-scale randomised controlled trials with long-term follow-up, there is currently no evidence available regarding the ability of these cardioprotective agents to prevent CHF or to improve survival for cancer patients. 

Dexrazoxane is an orphan drug that protects the heart against anthracycline-associated damage by chelating iron (which prevents the formation of DOX-iron complexes and subsequent ROS production) and/or by inhibiting and depleting Top2β enzymes [13,14,15]. Dexrazoxane is approved for adult patients with advanced or metastatic breast cancer who have already received a cumulative dose of anthracyclines equivalent to ≥300 mg/m^2^ DOX [11]. However, no specific therapies have been approved to prevent or treat anthracycline-associated cardiotoxicity in children or adolescents [16] and there is a clear need for additional therapies in adult cancer populations to reduce the risk of adverse cardiovascular events. 

Exercise is an emerging therapy for the treatment of anthracycline-associated cardiotoxicity. Studies in rodents have demonstrated that exercise is a potent cardioprotective stimulus against the damaging effects of DOX. Two recent systematic reviews of preclinical studies concluded that physical exercise interventions (i.e., forced treadmill running or voluntary freewheel running) mitigate DOX-induced impairments in fractional shortening (FS), a measure of systolic function [17,18]. Exercise can improve cardiac function regardless of whether the training intervention is performed prior to or concomitantly with and/or after DOX treatment, although exercise performed prior to DOX exposure tends to have a greater effect on preserving cardiac contractility [18]. Interestingly, even short-term (5 days) or a single bout (60 min) of aerobic exercise can provide a significant benefit in protecting the heart against DOX-induced damage [19,20]. These findings provide a strong evidence base for the implementation of both short- and long-term exercise interventions in clinical settings of anthracycline cardiotoxicity.

A handful of small clinical trials have reported beneficial effects of exercise on cardiopulmonary function in breast cancer patients undergoing anthracycline chemotherapy [21,22]. The recent Breast Cancer Randomised Exercise Intervention (BREXIT) trial involving 104 women with early-stage breast cancer provides strong evidence that exercise can improve cardiovascular health for breast cancer patients undergoing anthracycline chemotherapy [23]. In this randomised trial, patients were prescribed an individualised and progressive multimodal exercise intervention and followed for 12 months. The exercise intervention significantly increased the VO_2_ peak (a measure of functional capacity) and was associated with a higher cardiac output, stroke volume and LVEF. Notably, functional disability—an important prognostic marker—at 12 months was completely prevented in patients who adhered to the exercise program. 

In light of these promising clinical findings, we have reviewed the literature to identify cellular mechanisms by which exercise is likely to protect the heart in settings of anthracycline-associated cardiotoxicity. The protective effects of exercise are multifactorial (see Figure 1). Exerkines—factors that are secreted in response to exercise—can act on multiple cell types in the organ of origin or in distal organs, including the heart. Improved understanding of the cellular mechanisms by which exercise protects the heart in settings of both acute and chronic anthracycline-associated cardiotoxicity may inform clinical studies to optimise the timing, type and duration of exercise interventions, and may lead to the development of novel therapeutics for cardiotoxicity management and treatment. 

## 2. Oxidative Stress

### 2.1. Exercise Prevents Oxidation of Biological Molecules

Oxidative stress is a major contributor to DOX-associated cardiotoxicity. DOX generates free radicals via a one-electron reduction in the mitochondrial electron transport chain (ETC), namely complex I [24]. During DOX metabolism, oxidoreductases convert DOX to a semiquinone radical, inducing the production of reactive oxygen species (ROS) superoxide and hydrogen peroxide [25,26,27]. DOX-iron complexes reduce hydrogen peroxide to form hydroxyl radicals [28]. The presence of free radicals causes oxidative post-translational modification (PTM) of biological molecules (DNA, proteins, lipids and carbohydrates) which is implicated in the pathogenesis of numerous CVDs [29].

Protein and lipid oxidation following DOX administration is evident by increased cardiac protein carbonyl and malondialdehyde (MDA) [19,30,31,32,33]. Protein carbonylation (an irreversible PTM) occurs in response to oxidative stress. Carbonyl groups (aldehydes, ketones and lactams) are introduced into protein side chains, resulting in the loss of protein function. DOX-iron complexes are also known to induce lipid peroxidation, a process of oxidative degradation of lipid carbon–carbon double bonds, resulting in the formation of MDA as a byproduct [34]. Abnormal protein accumulation and cell/organelle membrane damage ultimately induces cell death. 

Marques-Aleixo et al., investigated the effects of aerobic exercise (12 weeks forced or voluntary wheel running) on DOX-induced protein carbonylation and lipid peroxidation in male rats [30]. Exercise performed concurrent with DOX therapy significantly attenuated myocardial protein carbonylation and decreased mitochondrial MDA levels. Similarly, Chicco et al., observed significant attenuation of left ventricular MDA with exercise preconditioning (8 weeks voluntary wheel running) in female rats, [31] and 3 weeks of exercise preconditioning or a single exercise bout prior to DOX-attenuated myocardial MDA levels in male rats [19,32]. These findings demonstrate that exercise performed either concurrent with or prior to DOX treatment can significantly attenuate DOX-induced protein carbonylation and lipid peroxidation. 

### 2.2. Exercise Upregulates Antioxidants

A mechanism by which the body protects against oxidative damage is via the upregulation of endogenous antioxidants. Superoxide dismutases (SOD) are enzymes that catalyse the dismutation (simultaneous oxidation and reduction) of superoxide to molecular oxygen and hydrogen peroxide [35]. Glutathione peroxidases (GPx) utilise glutathione (GSH) to enzymatically reduce hydrogen peroxide to water [36]. Similarly, catalase (CAT) breaks down two hydrogen peroxide molecules to produce water [37]. Thus, upregulation of endogenous antioxidants protects against ROS-induced damage. 

Numerous studies have investigated antioxidant responses to exercise performed either concurrent with or prior to DOX treatment. Exercise training (6–8 weeks forced or voluntary running) concurrent with DOX treatment increased cardiac SOD levels in both male and female rodents [30,38]. A longer running intervention (12 weeks, female rats) or swim training (8 weeks, sex not specified) also increased cardiac CAT levels [39,40] Chronic exercise preconditioning (3–14 weeks treadmill running) increased cardiac SOD levels in male rodents [32,33,41,42,43]. Interestingly, a short 5-day aerobic training intervention conducted prior to DOX treatment was also sufficient to increase cardiac SOD, GPx and CAT (male rats) [42]. Ascensao et al., published two studies evaluating the effects of 14 weeks of exercise preconditioning in male rodents, one utilising treadmill running and the other swimming [41,44]. The authors found that swim training significantly increased CAT but not SOD or GPx levels, whereas treadmill running was able to increase SOD activity. 

These findings highlight how variations in exercise regimes (type, timing, duration and intensity) induce varying degrees of cardioprotection against DOX-induced oxidative stress. Age and sex may also influence the antioxidant response to exercise. For example, 3 weeks of treadmill preconditioning led to higher SOD levels in young male rats compared to adult or old rats [34]. This is not surprising given that the free radical scavenging activity of endogenous antioxidants is known to decline with increasing age [45]. It remains unclear whether exercise training is of benefit for elderly individuals exposed to anthracyclines. An acute bout of strenuous exercise induced significant ROS production in senescent hearts from 25-month-old rats compared with hearts from 8-month-old rats, suggesting that high intensity exercise could potentially exacerbate oxidative stress in older individuals [46]. Further research comparing the efficacy of different exercise interventions in male and female animals, at different ages, may assist in the design of effective exercise regimes for specific patient populations. 

### 2.3. Exercise Maintains Cellular Protein Homeostasis

Heat shock proteins (HSPs), which are highly conserved chaperones, facilitate the restoration of misfolded proteins and prevention of protein denaturation and aggregation, thus contributing to cellular protection. HSPs are produced in response to various stressors, including exercise, which involves temperature fluctuations, free radical formation, energy depletion, pH alterations and transient hypoxia [47]. The 70-kDa family of HSPs (HSP70 and HSP72) has been the subject of numerous investigations because of its demonstrated function in defending the heart in settings of ischaemic reperfusion (I/R) injury [48,49,50]. In settings of DOX-induced cardiotoxicity, the role of exercise preconditioning on HSP induction and cardiomyocyte protection has been investigated in five studies [31,32,41,42,51]. All showed that exercise training (3–14 weeks forced or voluntary running, male and female rodents) increased the myocardial expression of HSP70/72. Kavazis et al., investigated if HSP72 is required to achieve protection against DOX-induced cardiotoxicity by exercising male rats under cold conditions (4°) to prevent the heat shock response and upregulation of HSP72 [42]. Exercise training increased the expression of antioxidants (SOD and GPx) and decreased ROS production and mitochondrial damage in rats trained at room temperature and in the cold, suggesting that these cardioprotective effects occur independently of HSP72 induction. Weeks et al., investigated Hsp70 expression and its involvement in mediating the cardioprotective effects of phosphoinositide 3-kinase (PI3K) p110α, a critical regulator of exercise-induced cardiac protection and physiological cardiac hypertrophy [52,53,54]. Cardiac Hsp70 expression was robustly increased by exercise and in transgenic mice with elevated PI3K activity. However, deletion of Hsp70 in PI3K transgenic mice had no effect on the degree of protection against pressure overload-induced remodelling, indicating that Hsp70 is not required for PI3K-mediated cardioprotection. Similarly, Bernardo et al., showed that HSP70 overexpression was unable to confer protection in a mouse model of atrial fibrillation (AF) or chronic HF [55]. Collectively, the findings of these studies suggest that exercise-induced increases in HSP expression alone are not sufficient to alleviate DOX-induced cardiotoxicity. 

## 3. Mitochondrial Adaptations

### 3.1. Exercise Preserves Mitochondrial Structure

Mitochondria are membrane-bound organelles that produce most of the energy required for the rhythmic contractile activity of the heart. Approximately 30% of cardiac volume is comprised of mitochondria [56]. The adult heart primarily relies on fatty acid oxidation (60–70%) to produce adenosine triphosphate (ATP) [57]. ATP synthesis occurs via a calcium-dependent process in the mitochondria known as oxidative phosphorylation (OXPHOS). The abundance and strategic positioning of mitochondria, under the sarcolemma and between myofilaments, aids in the efficient delivery of ATP required to sustain electrical and mechanical activities. As the main organelle responsible for energy production, mitochondria are vital in maintaining cardiac function. 

Structural damage of mitochondria has been observed in DOX-induced cardiotoxicity. Cardiomyocytes exposed to DOX exhibit significant mitochondrial ultrastructural alterations including swelling, vacuolisation and lack of intact cristae [58]. Wang et al., utilised transmission electron microscopy (TEM) to visualise mitochondrial ultrastructural differences in sedentary and exercised female mice treated with DOX [59]. Four injections of DOX over a 2-week period (10 mg/kg cumulative dose) led to abnormal mitochondria and vacuolisation in hearts of sedentary mice. These abnormalities were absent in hearts of mice that underwent exercise concurrent with DOX treatment, or for 12 weeks following the 2-week DOX treatment period. These findings demonstrate that exercise preserves mitochondrial structure in settings of DOX-induced cardiotoxicity. 

### 3.2. Exercise Decreases Susceptibility to MPTP Toxicity

**Figure 2:**
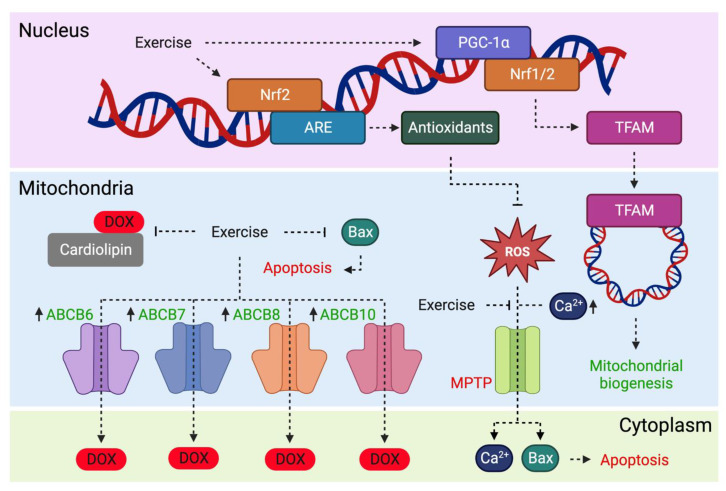
Effects of exercise on mitochondrial processes involved in doxorubicin (DOX)-associated cardiotoxicity. Exercise increases expression of peroxisome proliferator-activated receptor-γ coactivator 1α (PGC-1α) which binds to nuclear respiratory factors (Nrf1/2) and induces mitochondrial transcription factor A (TFAM) expression. Mitochondrial translocation of TFAM contributes to mitochondrial biogenesis. Exercise may reduce DOX accumulation in mitochondria by increasing expression of ATP-binding cassette (ABC) transporters (ABCB6, ABCB7, ABCB8 and ABCB10). Exercise increases Nrf2 expression, which is known to activate the antioxidant response element (ARE) and antioxidant gene expression. Antioxidants inhibit reactive oxygen species (ROS)-induced mitochondrial membrane permeability transition pore (MPTP) opening, a process that releases pro-apoptotic Bax and accumulates calcium (Ca^2+^). Image created with Biorender.com.

The mitochondrial permeability transition pore (MPTP) is a transmembrane protein channel in the inner mitochondrial membrane (IMM). In conditions of stress, such as calcium overload or oxidative stress brought on by DOX, the MPTP opens and releases solutes up to 1.5 kDa in size [60]. (See Figure 2). This induces mitochondrial swelling, uncoupling of OXPHOS and the release of proapoptotic factors and accumulated calcium, which ultimately induces apoptotic cell death [61]. A consequence of DOX therapy is increased sensitivity to MPTP opening [62]. Molecules capable of inhibiting MPTP opening, such as cyclosporine, have demonstrated the prevention of DOX-induced mitochondrial dysfunction and contractile impairment [62]. 

The potential role of exercise in decreasing the susceptibility of MPTP opening in cardiac mitochondria has been investigated in three studies [60,63,64]. A 60-min exercise intervention 24 h prior to DOX resulted in increased calcium tolerance and prevention of MPTP opening in male rats [60]. Similar findings were observed with 2 weeks of treadmill running in female rats [64]. Exercise preconditioning prevented DOX-induced increases in the maximal rate of pore opening, as well as a decrease in calcium retention capacity in female rats. Chronic exercise (12 weeks treadmill or voluntary wheel running) prevented cardiac MPTP opening susceptibility in male rats [63]. In summary, both forced and voluntary exercise can protect against mitochondrial dysfunction induced by DOX.

### 3.3. Exercise Induces Mitochondrial Biogenesis

Mitochondrial biogenesis is essential for maintaining cellular homeostasis. Peroxisome proliferator-activated receptor-γ coactivator (PGC)-1α is a critical regulator of mitochondrial biogenesis and is highly expressed in mitochondria-rich tissues, including the heart [65]. PGC-1α, encoded by *PPARGC1A*, coactivates transcription factors involved in OXPHOS and free radical detoxification [66]. For example, the expression of mitochondrial ETC subunits is regulated by PGC-1α binding to nuclear respiratory factors Nrf1 and Nrf2 [67,68]. Nrfs induce the expression of mitochondrial transcription factor A (Tfam), a protein required for the transcription and replication of mitochondrial DNA (mtDNA), thereby regulating mitochondrial biogenesis. (See Figure 2). PGC-1α deficiency blunts mitochondrial enzyme activity and reduces cardiac ATP, ultimately inducing cardiac dysfunction [69].

Targeting PGC-1α has therapeutic potential in preventing mitochondrial dysfunction. Melatonin, a potent antioxidant, was shown to inhibit mitochondrial dysfunction induced by DOX by upregulating PGC-1α and its downstream transcriptional factors (Nrf1, Tfam and uncoupling protein-2 (UCP-2)) [70]. Non-pharmacological interventions to upregulate PGC-1α expression in various tissues include fasting (liver), cold exposure (brown adipose tissue) and exercise (muscle) [71]. One study found that short-term exercise preconditioning prior to DOX significantly upregulated PGC-1α expression in male rats [72]. The authors suggested that the increase in PGC-1α suppressed myocardial FoxO1-induced atrophy, revealing a possible mechanism by which DOX exposure leads to a reduction in heart mass. Additionally, male rats that underwent forced treadmill and voluntary wheel running during DOX treatment had increased myocardial PGC-1α levels, although these findings were not statistically significant. This demonstrates that exercise preconditioning may protect mitochondria by upregulating mitochondrial biogenesis to account for DOX-induced mitochondrial degradation.

Nrf2 is also known to activate the nuclear antioxidant response element (ARE), thereby upregulating the expression of antioxidant genes (see Figure 2). Nrf2 activation with trimetazidine or exercise has been shown to confer cardiovascular benefits [73,74]. Nrf2 depletion in DOX-treated mice exacerbated cardiotoxicity, evident by increased oxidative stress and the aggregation of ubiquitinated proteins [75]. The potential of targeting Nrf2 signalling to protect cells against DOX-induced damage has been reviewed elsewhere [76]. An understanding of the transcriptional signalling pathways involved in exercise-mediated Nrf2 signalling has therapeutic potential in drug design, mimicking the cardioprotective effects of exercise for patients unable to participate. This may be particularly relevant in settings of acute cardiotoxicity where patients have strongly impaired cardiac function. 

### 3.4. Exercise Reduces Mitochondrial Accumulation of DOX

The IMM is comprised of a phospholipid bilayer containing complexes of the ETC involved in OXPHOS. Cardiolipin (CL) is a mitochondria-specific phospholipid found in the IMM. DOX accumulates in mitochondria due to the formation of DOX-CL complexes [77]. (See Figure 2). As a result, the close proximity of DOX to the ETC increases the susceptibility of redox cycling via complex I. Interestingly, DOX accumulates differentially in subsarcolemmal compared to intermyofibrillar mitochondria, with a greater affinity for the former [78]. The greater accumulation of DOX in subsarcolemmal mitochondria may reflect its proximity to capillaries or a greater abundance of cardiolipin in this mitochondrial population [79,80]. Nrf1 expression was significantly reduced in the intermyofibrillar population following DOX treatment, which was accompanied by a greater apoptotic and autophagic response [78].

Several studies have investigated changes in myocardial DOX accumulation with exercise. Lower myocardial DOX accumulation was observed with 2 weeks of exercise preconditioning in male and female rodents, with significantly lower levels in the mitochondrial but not the cytosolic fraction [64]. Jensen et al., compared forced and voluntary wheel running (10 weeks) on the accumulation of DOX in the left ventricle of female rats [81]. The authors found that DOX accumulation was ~50% lower in the treadmill group relative to time-matched sedentary controls at days 1 and 3 after receiving a DOX injection, and ≥60% lower at days 5 and 7. Voluntary wheel running was equally as efficacious at reducing DOX accumulation. It is thought that exercise preconditioning may lower mitochondrial DOX accumulation by regulating the expression of ATP-binding cassette (ABC) transporters. (See Figure 2). There are four ABC transporters located within mitochondria (ABCB6, ABCB7, ABCB8 and ABCB10) that serve to transport substrates across cell membranes [82]. Previous studies have shown that modifying ABC transporter expression can change the extent to which DOX accumulates in the heart [83,84]. Specifically, blockade of MRP1 induces significant myocardial accumulation of DOX and doxorubicinol (DOXoL) 24 h post-DOX administration, demonstrating enhanced cardiotoxicity in the absence of ABC transporters [84]. Morton et al., implemented a 2-week exercise preconditioning intervention and observed a significantly higher myocardial expression of the four ABC transporters in female rats [64]. Thus, it is likely that exercise shields the heart from DOX-induced injury by accelerating the rate of DOX clearance through increased expression of ABC transporters.

### 3.5. Exercise Prevents Mitochondrial-Mediated Apoptosis

Apoptosis is an irreversible form of programmed cell death, a key mechanism contributing to DOX-associated cardiotoxicity [85]. Cells undergoing apoptosis are characterised by cell shrinkage, DNA fragmentation and plasma membrane protrusions driving the formation of apoptotic bodies [86]. Phagocytosis removes apoptotic bodies to prevent spillover and damage to neighbouring cells. Apoptosis can be induced via the intrinsic (mitochondrial-mediated) or extrinsic (death receptor-mediated) signalling pathway. Apoptosis via the mitochondrial pathway is triggered by an apoptotic stimulus, such as oxidative stress, that induces proapoptotic protein activation. Proapoptotic Bax accumulates at the outer mitochondrial membrane (OMM) where it oligomerises and initiates membrane permeabilization [87]. Cytochrome c, an electron carrier found within mitochondria, binds to cytosolic apoptosis protease activating factor-1 (Apaf-1). This interaction induces the formation of a heptameric apoptosome, a protein complex driving the activation of effector caspases and ultimately inducing apoptosis. 

The role of exercise in preventing mitochondrial-mediated apoptosis has been investigated mostly in relation to the expression of apoptotic mediators and activation of caspases in male rodents. Treadmill running as a form of exercise preconditioning was evaluated in five studies [41,42,60,63,88]. One exercise session completed 24 h before DOX exposure significantly attenuated the DOX-induced rise in myocardial caspase 3, indicative of apoptosis inhibition [60]. A similar degree of protection was observed with short-term (5 days) and long-term (14 weeks) exercise preconditioning [41,42]. Long-term exercise preconditioning also significantly attenuated DOX-induced proapoptotic Bax induction [41]. High intensity interval training (HIIT) for 6 weeks significantly reduced the expression of Bax and caspase-6 [88]. Moreover, 12 weeks of forced and voluntary wheel running were equally as efficacious at attenuating Bax, caspase 3, 8 and 9 expression [65]. Chicco et al., investigated the effects of low intensity exercise training (LIET) over 2 weeks of DOX therapy and observed significantly reduced caspase 3 activity, ~20% below control levels [89]. Exercise postconditioning was also assessed to determine whether exercise following DOX therapy could protect against DOX-induced apoptosis [90]. The 4-week period of treadmill running post-treatment prevented the associated increase in cleaved caspase 3; however, no difference was observed in Bax expression with DOX or exercise. In summary, exercise performed prior to, during or after DOX therapy can prevent DOX-induced mitochondrial-mediated apoptosis. 

## 4. Cardiac Adaptations

### 4.1. Exercise Preserves Cardiomyocyte Ultrastructure

The cardiomyocyte cell population is responsible for heart contractility. Cardiomyocytes are composed of myofibrils that run parallel to each other, forming muscle fibres. Myofibrils are composed of repeating contractile units termed sarcomeres, defined by two consecutive Z lines. During cardiomyocyte contraction, the distance between the Z lines shorten. This is due to myosin and actin filaments sliding within the sarcomere. Specifically, actin filaments slide along myosin filaments as a result of actin-myosin cross-bridge cycling. The M line running through the middle of the sarcomere, composed of myomesin, and the Z lines on either side, function to hold the actin and myosin filaments in place. Impairment of sarcomeric structure and function is associated with diminished cardiac contraction (systolic dysfunction) and/or chamber filling (diastolic dysfunction). 

Sequeira et al., examined the effects of 10 mg/kg of DOX (cumulative) on the ventricular cardiomyocyte ultrastructure in female rats [39]. Cardiomyocytes from DOX-treated animals had disorganised myofibrils and sarcomeres, fragmented actin and myosin filaments and wavy Z lines with some areas showing an absence of Z lines. In contrast, rats that performed forced treadmill running throughout the course of the DOX treatment showed preserved myofibril integrity and improved sarcomeric organisation with reduced fragmentation of myofilaments. The data from this study suggest that aerobic exercise performed concurrent with DOX therapy is able to preserve the ultrastructural integrity of cardiomyocytes and ultimately preserve cardiomyocyte contractility. 

### 4.2. Exercise Prevents MHC Isoform Shifts

Alterations in the composition of sarcomeres may contribute to DOX-induced reductions in contractility. In humans, α-myosin heavy chain (MHC) is the predominant MHC isoform in atria, while β-MHC is the predominant isoform in the ventricles [91]. Smaller mammals, such as rodents, exhibit higher cardiac energetics and predominantly express the α-MHC isoform in the ventricles. Increasing the ratio of β-MHC to α-MHC reduces overall energy requirements but is associated with reduced contractility. In this context, the peak power output of cardiomyocytes exclusively expressing the β-MHC isoform was ~50% lower than cardiomyocytes expressing 12% α-MHC and 88% β-MHC [92]. 

It has previously been shown that DOX treatment upregulates β-MHC expression in the left ventricle of male rodents [93,94,95,96]. Exercise preconditioning for 10 weeks significantly attenuated the expression of β-MHC in male rats administered a DOX dose of 10 mg/kg [93]. The same group showed that DOX administered daily (1 mg/kg) for a period of 10 days increased β-MHC expression in the left ventricle. This response to DOX treatment was somewhat blunted in animals that underwent either forced or voluntary wheel running for 10 weeks prior to DOX [94]. Similar findings were reported in a rat model of resistance training [97,98]. Thus, exercise training may preserve cardiac contractility in part by maintaining a normal α-MHC/β-MHC ratio. 

### 4.3. Exercise Alleviates Fibrosis

Cardiac fibrosis is a term used to describe the pathological process of scar tissue formation and interstitial collagen deposition that can lead to myocardial stiffness and dysfunction. The extracellular matrix (ECM) describes the network of non-cellular fibrous and non-fibrous proteins contributing to the maintenance of tissue architecture. Collagen is the most abundant fibrous ECM protein, accounting up to 30% of total protein found in the human body [96]. The upregulation of collagen as a means of tissue repair can result in collagen accumulation and fibrosis formation. Clinically, anthracycline-based therapy has shown to increase the cardiac extracellular volume fraction (marker of fibrosis) as early as three months from treatment initiation [97,98]. Similarly, histological analyses of rodent hearts show extensive fibrosis induced by DOX therapy [39,59,99,100]. 

The anti-fibrotic effects of exercise performed concurrent with DOX therapy has been evaluated in four studies. Sequeira et al., reported significant myocardial fibrosis in female rats that received a DOX dose of 10 mg/kg (cumulative) [39]. Moderate aerobic exercise training prevented this DOX-induced increase in fibrosis, evident by preserved connective tissue volume density. Similarly, Wang et al., observed significant collagen deposition in female mice immediately after DOX therapy, which persisted for 12 weeks [59]. The authors did not observe any significant fibrosis in animals that underwent treadmill walking during DOX therapy, demonstrating that exercise can prevent the development of fibrosis in this setting. Interestingly, low-to-moderate aerobic exercise training was unable to prevent myocardial fibrosis or preserve LVEF in male mice that received a DOX dose of 25 mg/kg (cumulative) [99]. Yang et al., investigated signalling pathways contributing to DOX-induced fibrosis in male rats [100]. DOX induced the expression of fibrotic markers, namely transforming growth factor beta 1 (TGF-β1), connective tissue growth factor (CTGF), phosphorylated extracellular signal-regulated kinase (p-ERK) and specific protein 1 (Sp1). Moderate intensity aerobic exercise during DOX therapy was able to mitigate the expression of these fibrotic factors, thereby reducing fibrosis and preserving cardiac function. 

### 4.4. Exercise Preserves Cardiac Size

Cardiac atrophy, or a reduction in heart mass and size, has been reported in both children and adults undergoing DOX therapy [101,102]. In survivors of childhood cancer, DOX-associated cardiotoxicity can manifest as restrictive cardiomyopathy, evident 15 years post-exposure [101]. This condition is characterised by increased myocardial stiffness and reduced left ventricular dimensions. Among anthracycline-treated adults who develop cardiomyopathy, a dose-dependent reduction in left ventricular mass is observed [103,104]. The extent to which this is due to decreased cardiomyocyte size, as opposed to increased cardiomyocyte death, is not fully understood. 

DOX contributes to cardiac atrophy by disrupting the balance of protein synthesis and degradation [103,104]. The degradation of proteins is regulated by the ubiquitin proteasome system (UPS) and the autophagic-lysosomal pathway (ALP [105]. Ubiquitin-labelled proteins are degraded by the 26S proteosome, while ALP-mediated protein degradation involves the sequestration of proteins within lysosomes via microautophagy, macroautophagy or chaperone-mediated autophagy [105]. UPS-mediated protein degradation was observed in neonatal rat ventricular myocytes treated with a clinically-relevant concentration of DOX [103]. This may be a mechanism by which DOX contributes to cardiomyocyte atrophy, although cell size was not reported in the above study. DOX also stimulates autophagy in both cultured cardiomyocytes and in mouse hearts; however, impairments in lysosomal function block autophagic flux, leading to the accumulation of autolysosomes, ROS production and contractile dysfunction [104]. Four weeks of DOX treatment (10 mg/kg cumulative dose) increased the formation of autophagosomes in hearts of young female mice, an effect that was completely abolished with a 2-week aerobic exercise intervention [59]. The mechanisms by which exercise preserves autophagic signalling in settings of DOX-induced cardiotoxicity have not been extensively investigated, but exercise is known to alter autophagic gene expression in the heart [106] and 5 days of treadmill running prior to an acute DOX challenge (20 mg/kg, male rats) prevented DOX-induced increases in the expression of autophagy proteins (Beclin 1, ATG4, ATG7, ATG12 and LC3) and the lysosomal proteases cathepsin B and cathepsin L [107]. 

DOX-induced cardiotoxicity is characterised by a decrease in cardiomyocyte area and volume, which reduces the overall heart weight/body weight (HW/BW) ratio [39,99,108]. HW/BW ratio was 34% lower in male rats receiving DOX (4 mg/kg/week for 4 weeks) compared with controls, an effect that was prevented by exercise preconditioning [108]. Gomez-Santos et al., demonstrated significant reductions in left ventricular mass and cardiomyocyte area with 5 weeks of DOX treatment (5 mg/kg/week) in male mice [99]. Low-to-moderate aerobic exercise performed concurrent with DOX treatment blunted cardiac and cardiomyocyte atrophy in this model. Similarly, aerobic exercise concurrent with DOX treatment prevented the decline in cardiomyocyte volume density observed in female rats [39]. These studies demonstrate that exercise performed prior to or during DOX therapy can preserve cardiomyocyte size and prevent cardiac atrophy.

### 4.5. Exercise Preserves SERCA2A Activity

Impairment of calcium homeostasis has been implicated in the pathophysiology of DOX-associated cardiotoxicity [109]. Regulation of calcium homeostasis is controlled by the sarcoplasmic reticulum (SR), a specialised endoplasmic reticulum that surrounds the contractile myofilaments of muscle cells. The SR ATPase 2a (SERCA2a) calcium pump, expressed in cardiomyocytes and type I skeletal muscle, is a key protein that pumps calcium back into the SR, leading to myocyte relaxation. Thus, reduced activity or expression of SERCA2a contributes to impaired calcium handling, relaxation and contractility, while interventions that increase SERCA2a expression improve cardiac function in animal models of HF [110]. DOXoL, a metabolite of DOX, interacts directly with SERCA2a and is a potent inhibitor of SERCA2a activity and SR calcium uptake [111]. Interestingly, inhibition of calcium uptake by DOXoL was almost 100 times greater than that of the parent compound (DOX). 

The role of exercise in regulating SERCA2a expression in settings of DOX-induced cardiotoxicity has previously been investigated. Hydock et al., observed an ~80–90% decrease in SERCA2a expression in male rats receiving a daily DOX injection of 1 mg/kg for a period of 10 days [94]. Long-term exercise preconditioning (10 weeks forced or voluntary wheel running) protected rats from developing diastolic dysfunction in response to DOX exposure; however, this occurred independently of an increase in SERCA2a levels, indicating that other mechanisms may be contributing to the preservation of cardiac function in this setting. Lien et al., reported significant reductions in SERCA2a expression in male rats administered a single injection of DOX at a dose of 10 or 15 mg/kg [20]. Forced treadmill running or voluntary wheel running for 5 days prior to DOX injection attenuated DOX-induced reductions in SERCA2a expression, with treadmill running providing the most benefit. This was associated with improvements in diastolic and systolic function, for both exercise modalities. Dolinsky et al., reported that exercise training concurrent with DOX treatment (8 mg/kg/week) partially prevented DOX-induced reductions in SERCA2a expression in female mice [38]. Together, these studies reveal that exercise can preserve SERCA2a expression levels and thus attenuate DOX-induced cardiac dysfunction. 

## 5. Vascular Adaptations

### 5.1. Exercise Preserves Smooth Muscle Function

The main structural components of blood vessels are smooth muscle and endothelial cells. These cells modulate vascular tone (vasoconstriction and vasodilation) and facilitate autocrine, paracrine and endocrine functions that contribute to vascular homeostasis [112]. Oxidative stress can impair vascular function, resulting in vasodilation, inflammation and increased smooth muscle cell growth [113]. DOX induces vascular smooth muscle and endothelial dysfunction [59,114,115]. Gibson et al., investigated vascular function in isolated aortas from male rats exposed to DOX after a 14-week period of voluntary wheel running or no exercise intervention. DOX impaired the contractile response to phenylephrine (a vasoconstrictor), and the relaxation response to acetylcholine (a vasodilator) and sodium nitroprusside (an endothelium-independent dilator) in aortas pre-constricted with phenylephrine. Aortas from exercised animals were more sensitive to phenylephrine than aortas from sedentary animals, and exercise also improved the relaxation response to sodium nitroprusside but not acetylcholine, demonstrating that exercise protects vascular smooth muscle cell function from DOX-induced damage [114]. 

### 5.2. Exercise Preserves Endothelial Function

Vascular abnormalities were evident in a juvenile mouse model of DOX cardiotoxicity [59]. Young female mice treated with DOX (10 mg/kg cumulative dose) had a smaller number of blood vessels in cardiac cross-sections and a reduced ratio of α-smooth muscle actin to CD31-positive cells, suggestive of a reduced pericyte number. These effects were associated with a reduced mitral valve E/A ratio (a measure of diastolic blood flow). Daily treadmill walking performed throughout the 2-week DOX treatment period completely prevented the effects of DOX on these parameters. Lineage tracing experiments using bone marrow stem cells labelled with green fluorescent protein (GFP) revealed that in hearts damaged with DOX, exercise promotes the differentiation of bone marrow cells into pericytes and endothelial cells, and that this is associated with preservation of diastolic blood flow [115]. These findings suggest that vasculogenesis, i.e., the de novo formation of endothelial cells, contributes to cardiac vessel repair when damaged by DOX. This is in contrast to angiogenesis, where endothelial cells are derived from pre-existing vasculature. Additionally, the increased number of pericytes following exercise may contribute to the maintenance of an open lumen as well as cardiac tissue repair. These studies shed a light on the mechanisms by which exercise protects cardiac vasculature and ultimately protects against DOX-induced cardiotoxicity. 

## 6. Exerkines

### Exercise Releases Cardioprotective Factors

Exercise induces the release of factors, termed exerkines, from skeletal muscle (myokines), adipose (adipokines), brain (neurokines), liver (hepatokines), kidney (nephrokines), bone (osteokines) and the heart (cardiokines) [116]. Exerkines can act in an endocrine, paracrine and/or autocrine manner, aiding in inter-organ crosstalk [117]. Interleukin 6 (IL-6) is a myokine that is released from contracting skeletal muscle during exercise [118]. The pro- and anti-inflammatory activities of IL-6 are mediated via trans signalling (soluble receptors) and classical signalling (membrane-bound receptors) pathways, respectively [119]. Differential activation of these pathways may mediate protective or deleterious effects. McGinnin et al., demonstrated that IL-6 plays a role in mediating the cardioprotective effects of exercise preconditioning in a mouse model of ischemia/reperfusion injury [120]. Three days of treadmill exercise increased serum IL-6 and soluble IL-6 receptor (sIL-6R) levels, as well as myocardial expression of the IL-6 receptor (IL-6R). Wildtype mice that underwent exercise training prior to ischemia/reperfusion injury displayed fewer arrythmias and had smaller infarcts compared with sedentary controls [120]. In contrast, exercise was unable to protect against arrhythmia or necrosis in mice deficient for IL-6 [120]. IL-6-dependent inhibition of cardiomyocyte apoptosis during ischemia/reperfusion injury requires binding of circulating IL-6 to sIL-6R [121]. In the context of DOX cardiotoxicity, siRNA-mediated knockdown of IL-6 in H9C2 cells made cells more susceptible to DOX-induced apoptosis [122]. The findings from these studies suggest that IL-6 is a key player in mediating the protective effects of exercise, but its specific role in settings of anthracycline-associated cardiotoxicity requires further investigation. Further work is needed to determine if other exerkines, released in response to exercise, can elicit cardioprotective effects in settings of DOX-induced cardiotoxicity. 

Numerous groups have profiled serum and plasma obtained during and after various forms of exercise. This surge in profiling studies has led to the establishment of the Molecular Transducers of Physical Activity Consortium (MoTrPAC), which seeks to characterise the molecular changes occurring after acute and chronic exercise in both rodents and humans to uncover potential therapeutics to be used in the mitigation of disease [123]. Boström et al., discovered that PGC-1α regulates the expression of secretory gene products, one of which is fibronectin type III domain-containing protein 5 (FNDC5) [124]. Proteolytic cleavage of FNDC5, via exercise or PGC-1α upregulation, results in the secretion of soluble irisin [124]. Protective effects of irisin were observed in relation to obesity and glucose homeostasis [124]. Since its discovery in 2012, over 1500 studies have been published investigating the effects of irisin in various disease settings. A recent study investigated the role of irisin in cardiac perivascular fibrosis induced by DOX [125]. The authors observed significant reductions in serum and myocardial irisin with 4 weeks of DOX treatment (5 mg/kg/week) in male mice. Exercise training or treatment with recombinant irisin (1 mg/kg/d, 5 d/week) prior to and during DOX treatment led to significant increases in circulating irisin. Secretion of irisin was shown to mainly originate from cardiomyocytes, acting on endothelial cells via paracrine signalling to alleviate oxidative stress and reverse endothelial-to-mesenchymal transition via upregulating UCP-2. Moreover, both the exercise and irisin treatments significantly attenuated DOX-induced functional decline and partially alleviated perivascular fibrosis. 

## 7. Conclusions & Future Directions

**Figure 3:**
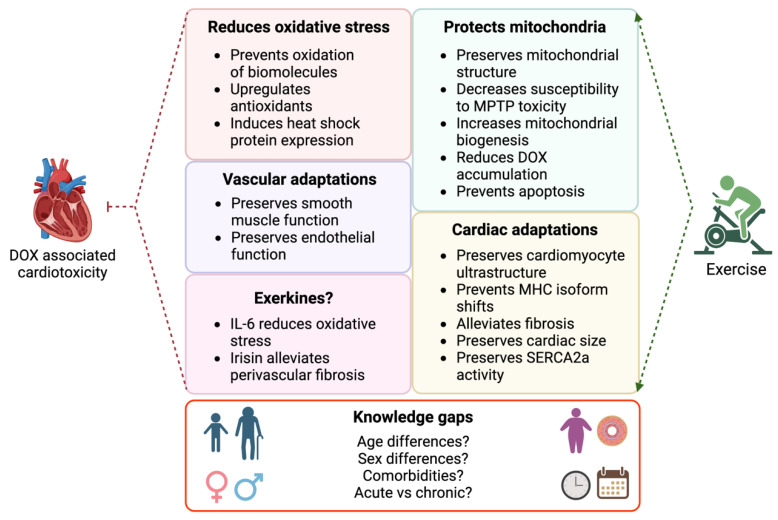
Mechanisms of DOX-associated cardiotoxicity and exercise-mediated cardiac protection, and knowledge gaps that need to be addressed by future research. Image created with Biorender.com.

There is extensive evidence from preclinical studies and accumulating evidence from clinical trials demonstrating that exercise is cardioprotective in settings of anthracycline-associated cardiotoxicity. Cellular and molecular mechanisms implicated in exercise-mediated cardioprotection include modulation of oxidative stress, protection of mitochondria, preservation of cardiac and vascular structure and function as well as exerkine-mediated effects. Animal models that more closely reflect the clinical situation will be important for delineating these mechanisms further (see Figure 3). For example, there is an absence of information regarding the effects of exercise on the hearts of anthracycline-treated tumour-bearing animals. An understanding of the cardiotoxicity–cancer relationship, and the protective effects of exercise, will help to identify biomarkers and therapeutics to prevent anthracycline-associated cardiotoxicity while preserving oncological efficacy. How comorbidities (e.g., obesity, hypertension, diabetes) influence this relationship should also be explored given the prevalence of these risk factors, particularly in older populations. Research is needed to evaluate the effect of exercise in both acute (short-term, high dosage) and chronic (long-term, low dosage) models of anthracycline-induced cardiotoxicity, and to determine the optimal type and dose of exercise in clinical populations with evidence of cardiotoxicity. We speculate that training will be beneficial in this context due to the established benefits of exercise training in patients with heart failure and reduced ejection fraction [126]. Testing drug combinations is also of clinical importance as patients typically undergo combination therapy (e.g., taxanes and anthracyclines). Finally, preclinical studies have mostly been conducted in otherwise healthy, male, adult rodents. Anthracyclines remain fundamental in the treatment of childhood cancers, with female sex a significant risk factor for anthracycline-associated cardiotoxicity in the paediatric cancer population [127]. Consideration of female sex hormones is also of clinical importance given that women represent the majority of breast cancer cases, and many receive endocrine therapy to slow or prevent the effects of oestrogen on tumour progression [11]. Further work to delineate the mechanisms of anthracycline-induced cardiotoxicity and exercise-mediated protection in male and female animals provides an opportunity to develop targeted therapies for male and female cancer patients.
